# Biological signaling pathways and potential mathematical network representations: biological discovery through optimization

**DOI:** 10.1002/cam4.1301

**Published:** 2018-04-10

**Authors:** Clara Isaza, Juan F. Rosas, Enery Lorenzo, Arlette Marrero, Cristina Ortiz, Michael R. Ortiz, Lynn Perez, Mauricio Cabrera‐Ríos

**Affiliations:** ^1^ Public Health Program Ponce Health Sciences University Ponce 00732‐7004 Puerto Rico; ^2^ Industrial Engineering Department The Applied Optimization Group University of Puerto Rico‐Mayagüez Mayagüez 00681‐9043 Puerto Rico; ^3^ Biology Department The Applied Optimization Group University of Puerto Rico‐Mayagüez Mayagüez 00681‐9043 Puerto Rico

## Abstract

Establishing the role that different genes play in the development of cancer is a daunting task. A step toward this end is the detection of genes that are important in the illness from high‐throughput biological experiments. Furthermore, it is safe to say that it is highly unlikely that these show expression changes independently, even with a list of potentially important genes. A biological signaling pathway is a more plausible underlying mechanism as favored in the literature. This work attempts to build a mathematical network problem through the analysis of microarray experiments. A preselection of genes is carried out with a multiple criteria optimization framework previously published by our research group 1. Afterward, application of the Traveling Salesperson Problem and Minimum Spanning Tree network optimization models are proposed to identify potential signaling pathways via the most correlated path among the genes of interest. Biological evidencing is provided to assess the effectiveness of the proposed methods. The capability of our analysis strategy is also demonstrated through the undertaking of meta‐analysis studies. Three important aspects are novel in this work: (1) our joint analyses of different groups of lung cancer states reveal new correlations, biologically evidenced, and previously undocumented; (2) computation of the correlation coefficients from expression differences leads to an effective use of network optimization methods; and (3) the methods yield mathematically optimal correlation structures: no other configuration is better correlated using the available information.

## Introduction

The study of cancer biomarkers is an important issue due to the role they play in the early detection, diagnosis, and prognosis of cancer. A biomarker can be defined as a characteristic that is objectively measured and evaluated as an indicator of normal biologic processes, pathogenic processes or pharmacologic responses to a therapeutic intervention 2. Studying biomarkers and their behavior has great potential to contribute to the discovery and understanding of the origin and evolution of cancer 3.

The analysis of high throughput experiments such as microarrays can lead to the identification of potential cancer biomarkers. Microarrays can be used to simultaneously analyze the expression of thousands of genes to select those that are differentially expressed. After selection, differentially expressed genes still go through further experimental validation in order to be confirmed as biomarkers.

Unlike other diseases such as cystic fibrosis 4 or muscular dystrophy 5, where mutations of one gene can cause disease, no one single gene “causes” cancer 6. Identifying potential cancer biomarkers plays a critical part in the understanding of cancer. Establishing how these genes possibly interact with one another and how these interactions could possibly contribute to the evolution of cancer is just as important. One possible and accepted way to better understand these interactions is to identify a signaling pathway among cancer biomarkers. A signaling pathway can be defined as the sequential interaction of products (such as proteins) of different genes, which cause an effect in the behavior of a cell. The abnormal activation of signaling pathways can lead to several different diseases, including cancer. Identifying and understanding these abnormal signaling pathways could possibly contribute to the diagnosis and treatment of cancer 7.

This work discusses the issue of identifying a potential signaling pathway among a list of potential lung cancer biomarkers through a network representation that leads to optimal configurations. The list of potential biomarkers is determined from microarray databases through the application of Multiple Criteria Optimization 8, a strategy proposed by our research group. The network optimization models initially proposed in this work to obtain potential signaling pathways among potential cancer biomarkers are the Traveling Salesperson Problem (TSP) 9 and the minimum spanning tree (MST) 10.

## Literature Review

There is an inherent difficulty in analyzing microarrays (and—omics in general) associated with the large size of these experiments. Microarray experiments simultaneously measure the expression levels of thousands of genes, generating large amounts of data. The analysis of these data present a challenge to biologists; new tools are needed to derive biological insight from these experiments, including signaling pathways 11, 12, 13, 14, 15.

Currently several experimental methods for determining signaling pathways exist. Signaling pathways, which according to Baxevanis and Ouellette involve many direct protein‐protein relationships, can be mapped using protein‐protein interaction detection methods such as copurification and nuclear magnetic resonance 16. Each one of these types of experiments requires time, expertise, and economic resources, providing opportunities for improvement. The methodology proposed in this article, based on network optimization methods, is capable of determining a set of global optimal solutions. This overcomes some of the limitations of experimental methods.

There are also different computational methods for the identification or analysis of signaling pathways 17. The following table lists software programs found during a recent literature search (Table 1), most of which can be also found through a PubMed search 18.

**Table 1:** Pathway analysis software 12

Tool	Method
ArrayXPath	Fisher exact test; Multiple testing correction
Pathway miner	Fisher exact test
Signaling pathway impact analysis	*P*‐value, false discovery rate
PathRanker	3M,HME3M
MAPPFinder	Standardized difference score (z) from hypergeometric distribution

The ArrayXPath is a web‐based tool for profile mapping and visualization of microarray gene expression from biological pathway resources 19. ArrayXPath automatically recognizes microarray probe identifiers by analyzing the statistical significance of the association between submitted data and maps in the pathway database 12. ArrayXPath applies Fisher's exact test to evaluate the statistical significance of the correlations. This software analyzes clusters of gene expression, and searches already existing pathway databases such as GenMAPP, Kyoto Encyclopedia of Genes and Genomes (KEGG), and BioCarta, among others. The methods proposed in this work do not require genes to be analyzed as clusters, and identify a potential pathway solution among previously identified over or under‐expressed genes highlighted for a disease of interest. The analyses proposed in this work are based on mathematical optimization, which serve as a contrast to the statistical approaches found in the literature, such as those in ArrayXPath.

Pathway Miner is another web‐based tool that mines gene associations and networks in biological pathway information. The tool permits the analysis and interpretation of pathway information and networks based on the association with databases, suitable for high‐throughput analysis of gene expression data 12. Pathway Miner provides two options to analyze genes in a dataset: (1) to search genes based on their associations in metabolic and/or cellular and regulatory pathways from pathway resources, and (2) perform a statistical test and rank significant pathways based on their *P*‐values from three different resources: KEGG, BioCarta, and GenMapp 20. Pathway Miner applies a one‐sided Fisher's exact test, as ArrayXPath does. Similar to several existing methods and software, Pathway Miner relies on statistical procedures. The methodologies presented in this article, in contrast, are of deterministic nature.

Signaling pathway impact analysis (SPIA) is a software tool used to measure the actual perturbation on a given pathway under a particular condition 21. This package provides a technique for pathway analysis based on combination of two types of evidence, the over‐representation of differently expressed genes and the perturbation of the pathway as measured by expression changes. For each pathway, a *P*‐value is calculated. With the assumption that the number of differentially expressed (NDE) genes in a pathway follows a hyper‐geometric distribution, the probability of the number of differentially expressed (P_NDE_) genes in the given pathway is calculated. The second probability, probability of pathway perturbation (P_PERT_)_,_ is calculated based on the estimated amount of perturbation in each pathway due to the differential expression of the input gene list 12. This differential expression is calculated by subtracting the expression levels between all the control samples from all case samples for each particular gene. SPIA and its probabilistic capability could benefit from the deterministic optimal solution found by this work's proposed method for comparison or referencing purposes.

PathRanker is a software tool that can identify genetic pathways that dictate the response of metabolic networks to specific experimental conditions 22. Initially, PathRanker uses a nonparametric pathway extraction method to identify the most correlated paths through a metabolic network. Then, it extracts the defining structure within these top‐ranked pathways using both Markov clustering and classification algorithms. Furthermore, detailed node and edge annotations are defined, which enables tracking of each pathway, not only with respect to its genetic dependencies, but also for an analysis of the interacting reactions, compounds, and KEGG subnetworks 22. PathRanker relies on probabilistic clustering, to which a deterministic optimal solution might add a useful point of comparison as well.

MAPPFinder is another software that can dynamically link gene‐expression data to Gene Ontology (GO) 23 hierarchies. MAPPFinder calculates the percentages of genes measured that meet a user‐defined criterion 11. Such criterion is required for each specific GO node, and for the cumulative total of the number of genes meeting the criterion in a parent GO term combined with all its children, giving a complete picture of the number of genes associated with a particular GO term. Using this percentage and a z‐score, the user ranks the GO terms by their relative amounts of gene‐expression changes 11. As mentioned before, MAPPFinder requires a user‐defined criterion, which can be very subjective and possibly affect the convergence of the results. These issues might be circumvented by the methods like those proposed in this work.

The literature review presented here provides evidence for an opportunity to create a deterministic optimization‐driven approach to the construction of signaling path proxies, even as a way to contrast results from the stochastic/statistic approaches already available. As illustrated in the following sections, the optimization‐driven approach is capable of finding lists of important genes, as well as the most correlated path among them, without the need for the user to adjust parameters that influence the output, thus providing objectivity and repeatability. This characteristic sharply differs from that in artificial intelligence and purely‐statistic procedures, where significance areas, thresholds, assumed underlying distributions, and weights, among other factors, are decisions that importantly affect the results and that are left for the user to manipulate.

## Methodology Background

### Traveling salesman problem

The Traveling Salesman Problem is one the most famous combinatorial optimization problems 24. In its most common interpretation, the TSP tries to construct the shortest tour through *n* cities 25, for a salesperson to visit, usually going back to a preselected base city 26. In other words the TSP consists of an optimization problem that searches for a cyclic sequence within a network that minimizes a certain measure such as total cost or total distance.

**Figure 1:**
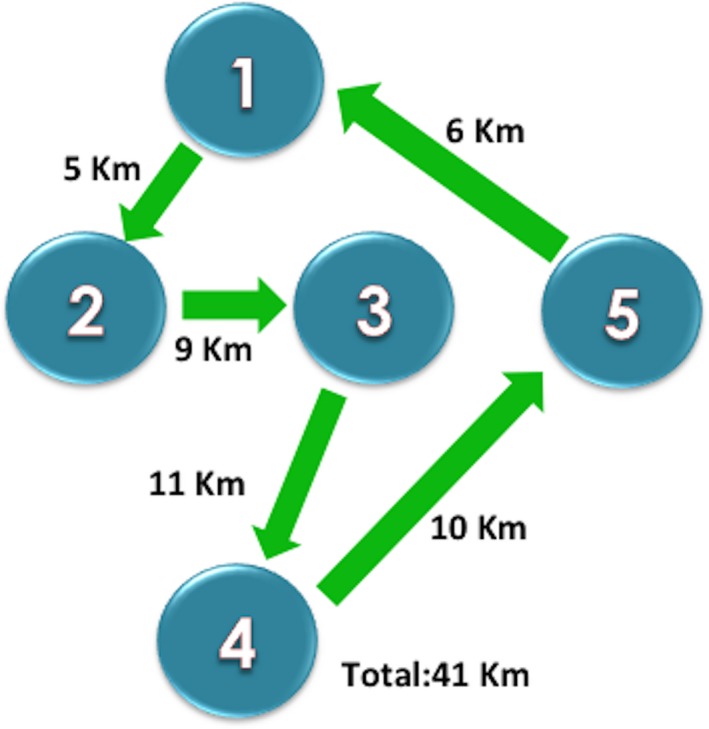
Example of the application of the traveling salesperson problem.

As an example, take the network presented in Figure 1 . A salesperson has to travel from city 1 through each city exactly once, and return home to city 1. If the objective was to obtain a tour that minimizes the total distances, that is a cycle with minimum total distance, there would be total of (5‐1)! = 24 possible cycles 27.

The TSP can be modeled as a network optimization problem. Consider that *c*_*ij*_ represents the cost of traveling from city *i* to city *j* and let *y*_*ij*_ be a binary variable, indicating whether or not the salesperson travels from city *i* to city *j*. In addition, let us define flow variables *x*_*ij*_ on each of the *m* arcs (*i,j*) and assume that the salesperson has *n*‐1 units available at node 1 (supply), which is arbitrarily selected as a “source node,” and the salesperson must deliver 1 unit to each of the other nodes or vertices (demand) [1]. The model is as follows [1]:

(1) $$\text{Minimize}\sum_{(ij)\in A}c_{\mathrm{ij}}y_{{}_{\mathrm{ij}}}$$

(2) $$\sum_{1\leq j\leq n}y_{\mathrm{ij}}=1\;\forall i$$

(3) $$\sum_{1\leq i\leq n}y_{\mathrm{ij}}=1\;\forall j$$

(4) Nx=b

(5) $$x_{\mathrm{ij}}\leq \left(n-1\right)y_{\mathrm{ij}}\;\forall \left(i,j\right)\in A$$

(6) $$x_{\mathrm{ij}}\geq 0\;\forall \left(i,j\right)\in A$$

(7) $$y_{\mathrm{ij}}=0\;\text{or}\;1\forall \left(i,j\right)\in A$$

Let A be the set of all arcs (*i,j*)*,* then let A’ = {(i,j):*y*ij = 0} that is the arcs forming in a solution to the previous model, and let A’’ = {(i,j):*x*ij > 0}, that is the arcs with nonnegative flow in a solution to the previous model. The problem tries to minimize the objective function representing the total cost of a solution in Equation (1). The constraints ((4) and (3)) imply that exactly one arc of *A’* leaves and enters any node *i*; therefore, *A’* is the union of node disjoint cycles containing all of the nodes. In general, any integer solution satisfying (eq. (4)) and (3)) will be union of disjoint cycles; if any such solution contains more than once cycle, they are referred to as subtours, as they pass through only a subset of nodes [1].

In constraint (4) *Ɲ* is a n × m matrix, called the *node‐arc incidence matrix* of the minimum cost flow problem. Each column $N_{\mathrm{ij}}$ in the matrix corresponds to the flow variable *x*_*ij*,_ in (4) all of them contained in the m × 1 vector *x*_._ The column $N_{\mathrm{ij}}$ has a value of +1 in the *ith* row to indicate incoming flow, and a value of −1 in the *jth* row to indicate outgoing flow; the rest of its entries are zero. The right hand side vector *b* with dimensions n*x*1 contains positive values to indicate supply in particular nodes, negative values to indicate demand in particular nodes, or zeroes in transshipment nodes. Constraint (4) ensures that *A”* is connected as we need to send 1 unit of flow from node 1 to every other node via the arcs in *A”*. The forcing constraints (5) imply that *A”* is a subset *A.’* These conditions imply that the arc set *A’* is connected and thus cannot contain subtours 9.

### Minimum spanning tree

The minimum spanning tree considers an undirected and connected network, where the given information includes some measure of the positive length (e.g., distance, cost, time, etc.) associated to each link 10, 28. The MST involves choosing a set of links that have the shortest total length among all sets of links that ensure that the chosen links provide a path between each pair of nodes 26.

**Figure 2:**
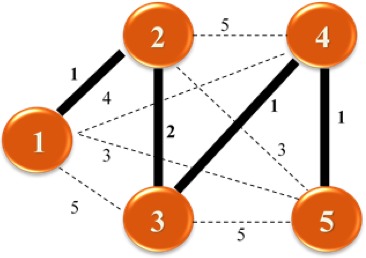
Example of a minimum spanning tree.

An example of the MST is presented in Figure 2 where we have five nodes of a network with their potential links and the positive length for each if it is inserted into the network. Enough links must be inserted to satisfy the requirement that there is a path between every pair of nodes. The objective is to satisfy this requirement while at the same time minimizing the total length of the links inserted into the network. In the example in Figure 2 the solution is highlighted by the darker and thicker lines. In the case of a spanning tree the total number of possible solutions con be calculated with Cayley's formula *n* ^*n*‐2^ where *n* is the number of nodes in the graph 29. In this particular example there is a total of 5^5‐2^ = 125 possible solutions

## Methodology

In this work the TSP and the MST are the methods proposed to obtain an optimal proxy for a signaling pathway. In order to begin to apply these methods however, the first step is to identify genes of interest based on the differences in expression when comparing control and cancer tissues. Multiple Criteria Optimization (MCO) based on Pareto conditions, as described in the works of Lorenzo et al. 30 and Camacho et al. 31, is used for this purpose. An MCO program developed in MatLab 31 is applied. The proposed network models are applied to structure the list of selected genes. The networks use statistical linear relationships between gene expression changes as measured by Pearson correlation between pairs 32, 33, 34.

Statistical correlation can be defined as a measure of the coordinated behavior between two random variables. It measures the strength or degree of association between two variables, say *X* and *Y*. Linear correlation values range from −1 to +1. The correlation can be considered more intense the closer the linear correlation values are to either +1 or −1. If we have a series of *n* measurements of *X* and *Y* written as *x*_*i*_ and *y*_i_ where *i *= 1,2…*n,* then the sample correlation coefficient can be used to estimate the Pearson correlation *ρ* between *X* and *Y* as follows:

(8) $$\rho_{{}_{\mathrm{xy}}}=\frac{\sum_{i=1}^{n}\left(x_{i}-\overset{¯}{x}\right)\left(y_{i}-\overset{¯}{y}\right)}{\left(n-1\right)s_{x}s_{y}}=\frac{\sum_{i=1}^{n}\left(x_{i}-\overset{¯}{x}\right)\left(y_{i}-\overset{¯}{y}\right)}{\sqrt{{\sum_{i-1}^{n}\left(x_{i}-\overset{¯}{x}\right)}^{2}\sum_{i-1}^{n}{\left(y_{i}-\overset{¯}{y}\right)}^{2}}}$$

where $\overset{¯}{x}$ and $\overset{¯}{y}$ are the sample means of *X* and *Y*, and *s*_*x*_ and *s*_*y*_ are the sample standard deviations of *X* and *Y* 35, 36.

**Figure 3:**
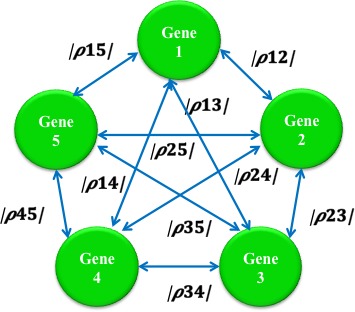
Representation of a potential sequence of a signaling pathway.

In this work, as a first approximation, the linear correlations that can be observed among a list of genes considered to be potential biomarkers are used as a base to construct networks such as the one presented in Figure 3.

As shown in Figure 3 there are many possible sequences that can be followed in order to discover a potential signaling pathway, even from a small list of genes. In this example, for five genes there can be (5‐1)! = 24 possible solutions. The number of possible solutions grows exponentially as more genes are included. The TSP can serve to support the discovery of a signaling pathway among a list of genes of interest with efficiency and efficacy since, if solved optimally, it will arrive at the most correlated cycle path.

### Traveling salesperson problem

The TSP can be used to discover a potential signaling pathway from a network of genes, by identifying a sequence that maximizes the linear correlation among the genes 30. The cyclic nature of the TSP solution would represent the feedback mechanisms present in gene product interactions. The rationale behind using a connecting model for the gene expression changes is that in biology nothing works in isolation. Every action in a cell or organism has an origin (coded information or stimuli) and will produce a response in another cell component. To maintain control over their internal conditions, cells coordinate their gene expression changes. Cells also have evolved to waste little energy, which is why this work proposes optimization models based on the coordinated gene expression changes as proxies for pathways.

For the TSP, a list of potential biomarkers of interest must first be identified. The list was obtained using MCO 8. In short, MCO aims to identify the best compromising solutions from a set of possible solutions characterized by at least two possible performance measures in conflict. The analysis of the selected lung cancer microarray database was modeled as an MCO problem. Database GDS3257, which was selected for this work contained readings for 22,283 genes and 107 samples, where 49 are control and 58 cancerous tissue samples 37.

**Figure 4:**
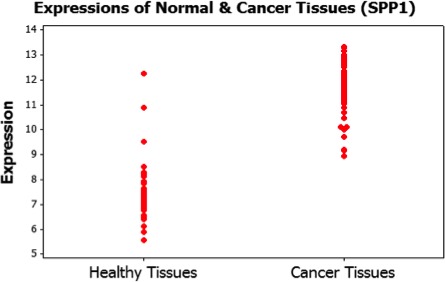
Representation of the expression differences in normal and cancer tissues.

Once the potential biomarkers were established, the next step was to calculate the differences in genetic expression for each gene. This was done by comparing all of its control samples against all of its cancer samples. Figure 4 graphically exhibits the differences in genetic expression in the case of the SPP1 gene, where each point is the level of expression for either a control or cancer tissue. SPP1 was identified as a potential lung cancer biomarker utilizing multiple criteria optimization based on Pareto optimality conditions.

**Figure 5:**
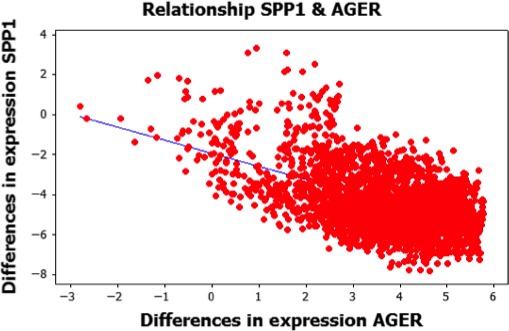
Example of correlation between potential biomarkers.

The genetic expression differences in all genes considered to be potential biomarkers were used to calculate the linear correlations among each pair. The values of the differences serve as input for calculating the linear correlations between all genes being considered (see eq. (8)). Figure 5 is a representation of the correlation between genes SPP1 and AGER based on their differences in genetic expression between control and lung cancer tissues. Each point in Figure 5 is graphed according to the difference in genetic expression between a particular pair of control and lung cancer tissue sample for SPP1 and AGER. Let us assume *d*_*1*_ represents the difference in genetic expression between a particular pair of control and lung cancer tissue samples for SPP1, and *d*_*2*_ is the difference in genetic expression between the same pair of tissue samples for AGER. Each point can be considered to be (*d*_*1*_ *, d*_*2*_).

The linear correlations between all genes of interest are calculated using Equation (8). The linear correlation values obtained can be associated with the arcs in a network model, such as the one in Figure 3. The absolute values of these linear correlations are used in order to obtain a sequence that maximizes the intensity of the linear correlations of the analyzed genes. Once the linear correlation values for all the genes are obtained, they are used to construct a matrix such as the one in Table 2. The 11 genes listed in Table 2 are potential lung biomarkers identified through MCO.

**Table 2:** Matrix of absolute values of pairwise gene correlations

	AGAR	SFTPC	TMEM100	FABP4	SPP1	WIF1	COL11A1	CYP4B1	FCN3	ADH1B	CLDN18
AGAR	0	0.643	0.436	0.515	0.531	0.496	0.363	0.348	0.545	0.364	0.567
SFTPC	0.643	0	0.373	0.410	0.410	0.570	0.432	0.550	0.441	0.544	0.550
TMEM100	0.436	0.373	0	0.305	0.270	0.226	0.159	0.239	0.600	0.433	0.319
FABP4	0.515	0.410	0.305	0	0.317	0.257	0.070	0.310	0.201	0.384	0.407
SPP1	0.531	0.410	0.270	0.270	0	0.373	0.375	0.155	0.277	0.273	0.462
WIF1	0.496	0.570	0.226	0.226	0.257	0	0.241	0.390	0.371	0.470	0.446
COL11A1	0.363	0.432	0.159	0.159	0.079	0.241	0	0.236	0.326	0.253	0.265
CYP4B1	0.348	0.550	0.239	0.239	0.319	0.390	0.236	0	0.264	0.497	0.239
FCN3	0.545	0.441	0.600	0.600	0.201	0.371	0.326	0.264	0	0.312	0.370
ADH1B	0.364	0.544	0.433	0.433	0.384	0.470	0.253	0.497	0.312	0	0.404
CLDN18	0.567	0.550	0.319	0.407	0.462	0.446	0.265	0.239	0.370	0.404	0

This matrix serves as input for the MCO program in MatLab (developed by our collaborator J. Rodríguez). The software is used to obtain an optimal sequence maximizing linear correlations among the eleven genes considered as potential biomarkers.

### Minimum spanning tree

The TSP has the somewhat restrictive assumption that a signaling pathway behaves as a tour. The MST is an alternative method that results in a noncyclic structure. The MST allows representation of groups of coordinated gene expression changes that may be independent from each other. The MST does not consider feedback because it is an open structure. It was deemed convenient to contrast these models as they have different advantages and could provide complementary information. The MST, as described in Section “Minimum Spanning Tree”, is applied to develop a signaling pathway proxy from a list of genes identified as potential biomarkers (see Table 3). Parting from the same matrix developed from the linear correlations of the genes of interest (see Table 2) the MST results in a minimal tree with the largest correlation among the nodes of interest.

**Table 3:** List of potential lung cancer biomarkers

Potential biomarkers	
AGER	COL11A1
SFTPC	CYP4B1
TMEM100	FCN3
FABP4	ADH1B
SPP1	CLDN18
WIF1	

## Lung Cancer Signaling Pathways

### Case study: lung cancer

According to the National Cancer Institute, lung cancer is the second most common type of cancer and the primary cause of cancer‐related death in both men and women in the United States. Also based on analysis conducted by this institution, an estimated $11.9 billion were spent on lung cancer care in 2014 38, 39.

**Figure 6:**
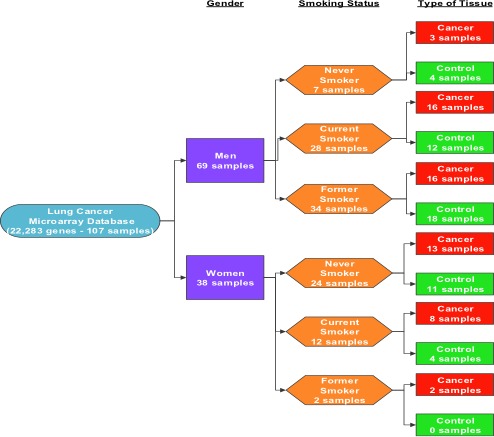
Representation organization of database (GDS3257).

As detailed previously, the GDS3257 database selected for this work was first reported by 37. It contains 107 samples, where 49 are control and 58 cancerous tissue samples. The subjects involved in this study ranged from the ages of 44–79 years old and had histologically confirmed primary lung adenocarcinoma, stages I–IV. The database also has detailed smoking and medical history information, which allows for division of the total of 107 samples into one of three groups: no history of smoking, former smokers, and current smokers (see Fig. 6). Each of these samples show the measured relative expression of a total of 22,283 genes.

TSP and MST were applied to identify potential signaling pathways from the list of eleven potential biomarkers for lung cancer obtained using MCO.

### Traveling salesperson problem

**Figure 7:**
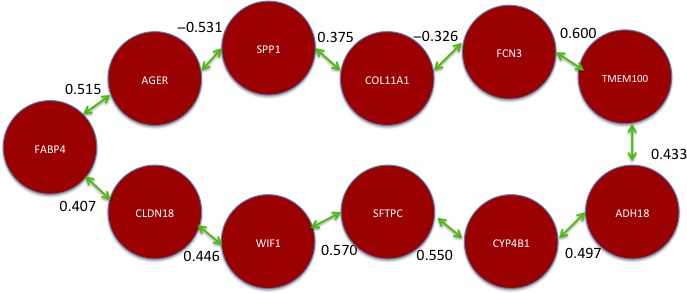
Optimal correlation cycle among 11 potential biomarkers obtained with Traveling Salesperson Problem.

By applying the TSP formulation, a sequence that maximizes the linear correlations from a large number of possible solutions was obtained using the methodology described by Lorenzo et al., 30. Figure 7 shows the results.

This sequence was obtained from the (11‐1)! ≈ 3.6 million possible solutions. It represents a solution that maximizes the linear correlations between the expression changes of these 11 genes. This result shows the potential of this work: being able to obtain an optimal cycling path from thousands (or millions) of possible solutions. It must be clarified that because this is an observational analysis of a microarray database, causality or directionality cannot be established at this point.

### Minimum spanning tree

**Figure 8:**
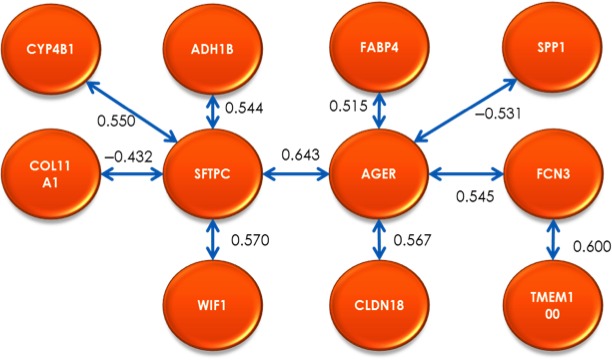
Optimal correlation walk among 11 potential biomarkers obtained with minimum spanning tree.

As an alternative method to the TSP, the MST was applied to obtain a tree that maximizes the linear correlations of the eleven genes that were identified previously. The resulting structure is presented in Figure 8.

The solutions obtained with the TSP and MST requires biological verification. There are scientific reports for some of the correlation links in the resulting models that will be discussed in Section “Biological Evidence and Validation of Proposed Methodology”.

Utilizing the combination of methodologies of MCO to determine a list of potential biomarkers from databases, and subsequently employing the TSP and MST to identify possibly signaling pathways from the genes of interest, could serve as a viable alternative to existing methods to provide an original analysis pipeline completely driven by optimization procedures.

### Meta‐analysis

This section describes the first steps taken to carry out a meta‐analysis in order to identify signaling pathways of interest from the different groups previously described in Section “Case Study: lung cancer” between control and cancer tissues groups within GDS3257 database. These groups include: 16 samples Cancer Never‐smoker (CNS) versus 24 samples Cancer Current Smoker (CCS), 16 samples Healthy Current Smoker (HCS) versus 24 samples CCS, 16 samples HCS versus 16 samples CNS, 15 samples Healthy Nonsmoker (HNS) versus 24 samples CCS, 15 samples HNS versus 16 samples CNS, and 15 samples HNS versus 16 samples HCS.

Initially, MCO was applied to identify genes with high variations in their relative expression levels when utilizing two performance measures, the absolute values of the differences between means and median as described in reference 31.

**Figure 9:**
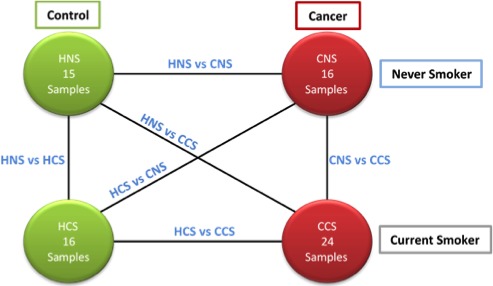
Representation of six analyses between four conditions in microarray database GDS3257.

The following step was to apply the TSP and MST optimization methods to identify potential signaling pathways. For each group comparison the TSP and MST were applied and a signaling pathway was obtained. Figure 9 represents the group comparisons conducted.

**Figure 10:**
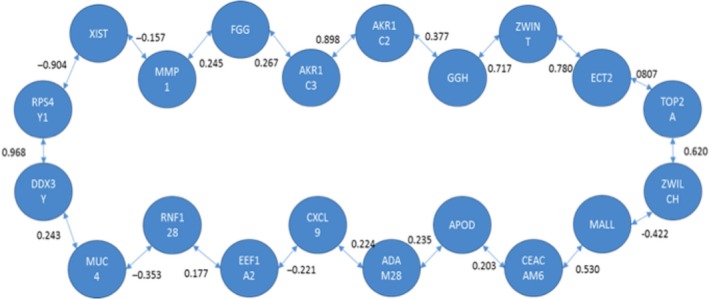
Optimal correlation tour using the traveling salesperson problem formulation (cancer never‐smoker vs. cancer current smoker).

**Figure 11:**
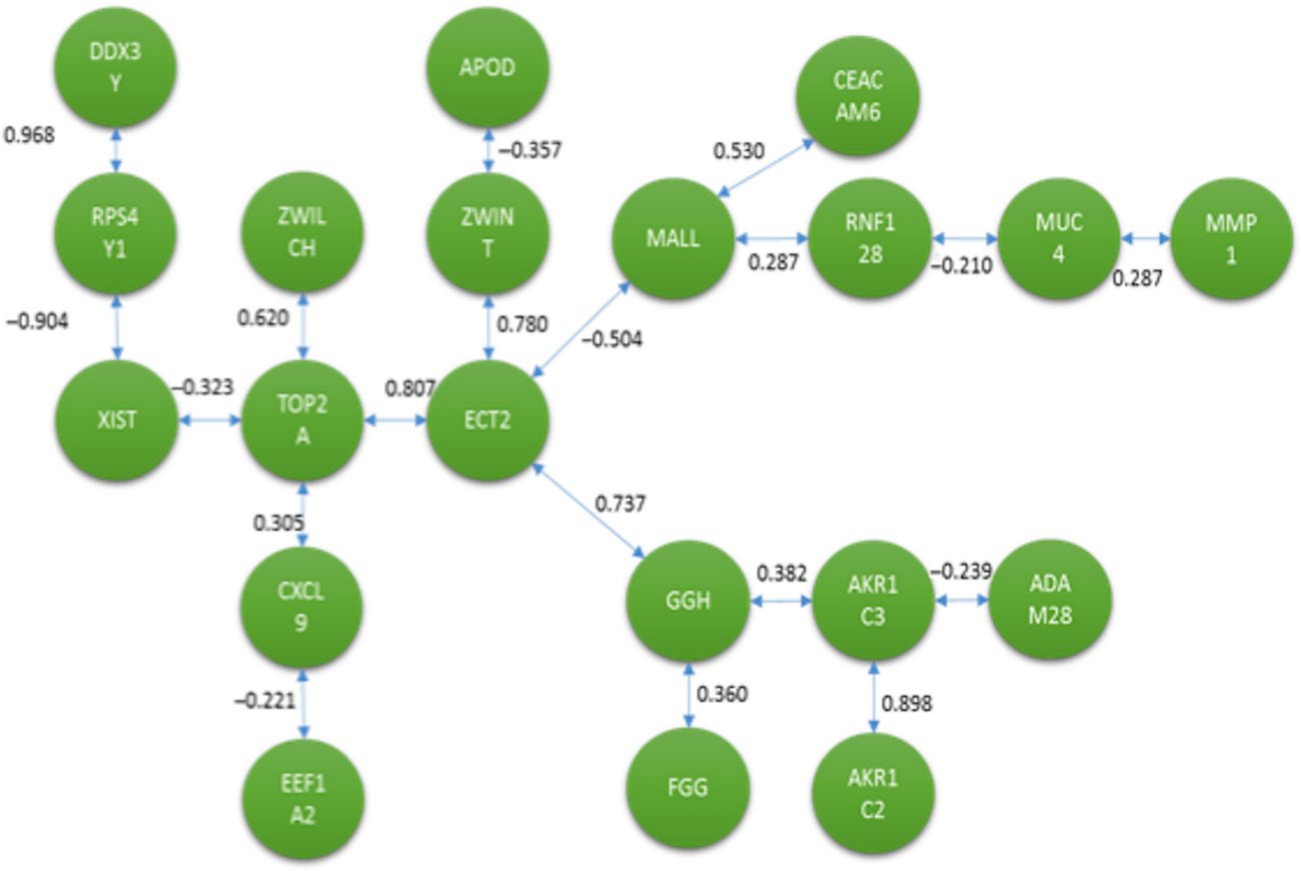
Optimal correlation tree using the minimum spanning tree formulation (cancer never‐smoker vs. cancer current smoker).

Both methods were applied to identify a signaling pathway when analyzing CNS versus CCS. Once MCO was conducted to determine the genes with the largest changes in relative expression, a total of 20 genes were identified as genes of interest for this particular comparison. The resulting network solution when applying the TSP and MST methods are represented by Figures 10 and 11 respectively.

Several similarities exist between both networks’ solutions that are potentially interesting from a biological perspective. These two networks are the best solution from a total of (20‐1)! and 20^20‐2^ possible configurations for the TSP and MST respectively.

**Figure 12:**
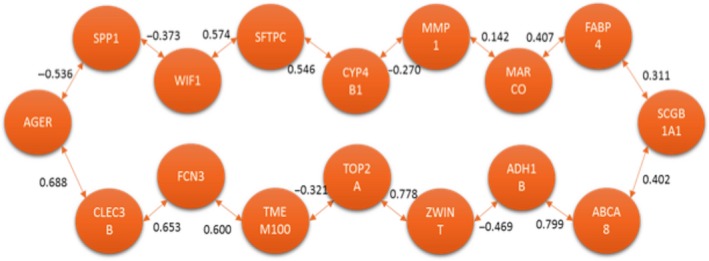
Optimal correlation tour using the traveling salesperson problem formulation (healthy current smoker vs. cancer current smoker).

**Figure 13:**
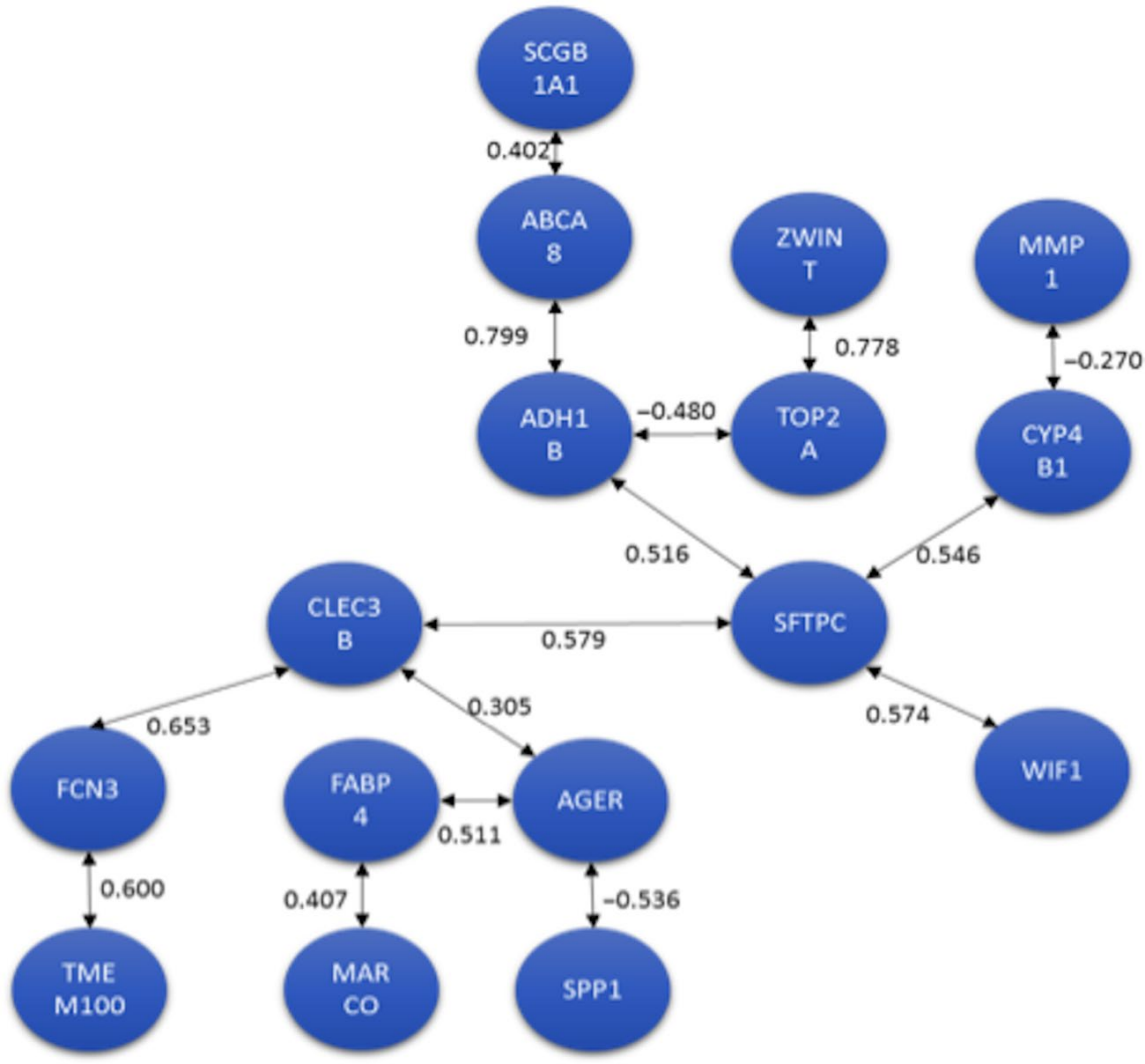
Optimal correlation tree using the minimum spanning tree formulation (healthy current smoker vs. cancer current smoker).

The next comparison conducted was for HCS versus CCS. The MCO solution produced a list of 16 genes. The same analysis described before was done. Figures 12 and 13 are the graphical representations of the solutions obtained for each method respectively. In these solutions, as for the previous comparisons, there are notable similarities.

**Figure 14:**
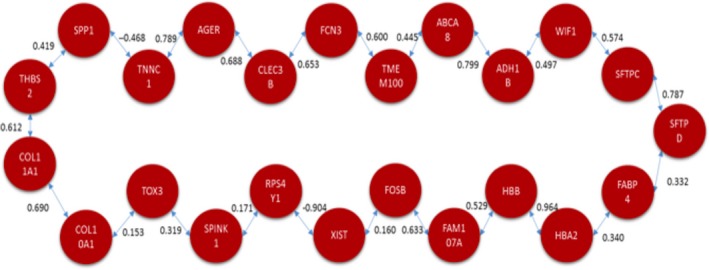
Optimal correlation tour using the traveling salesperson problem formulation (healthy current smoker vs. cancer never‐smoker).

**Figure 15:**
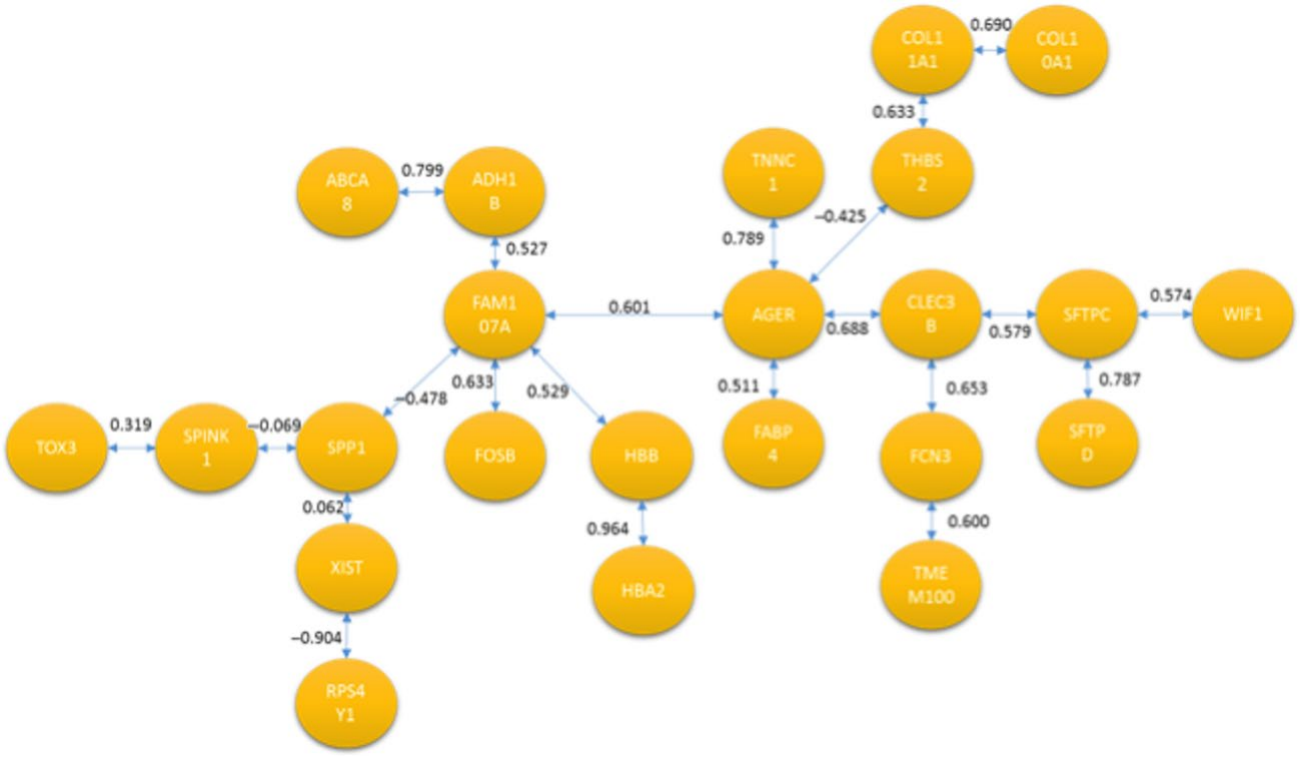
Optimal correlation tree using the minimum spanning tree formulation (healthy current smoker vs. cancer never‐smoker).

The third analysis was the comparison of HCS versus CNS, where a total of 23 genes were considered when carrying out the TSP and MST methods. The resulting signaling pathways are presented in Figures 14 and 15.

**Figure 16:**
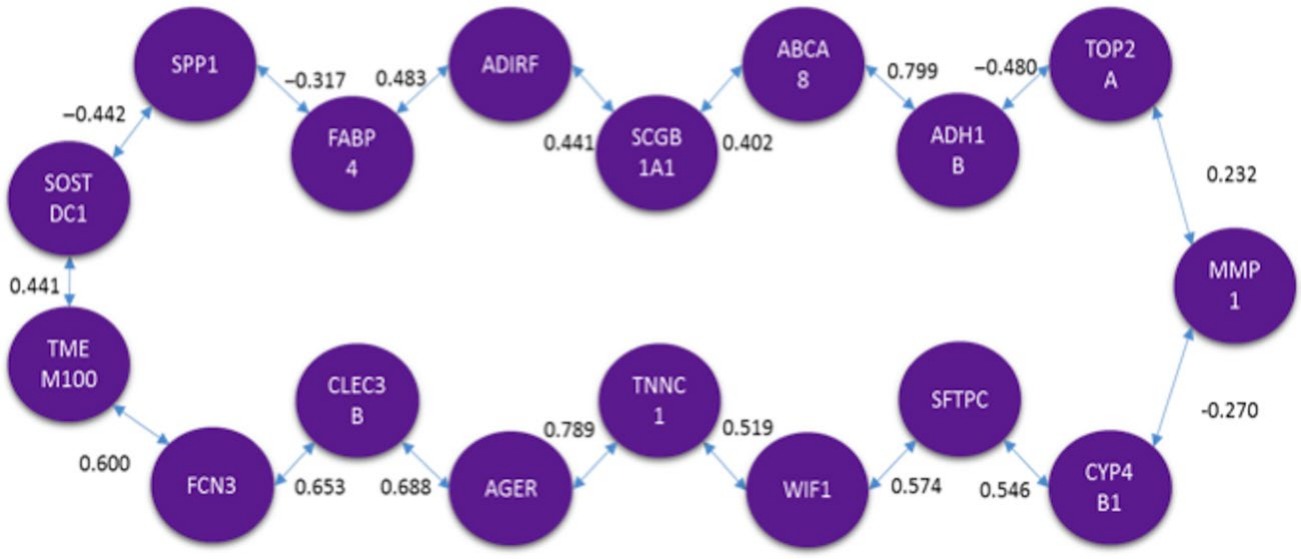
Optimal correlation tour using the traveling salesperson problem formulation (healthy nonsmoker vs. cancer current smoker).

**Figure 17:**
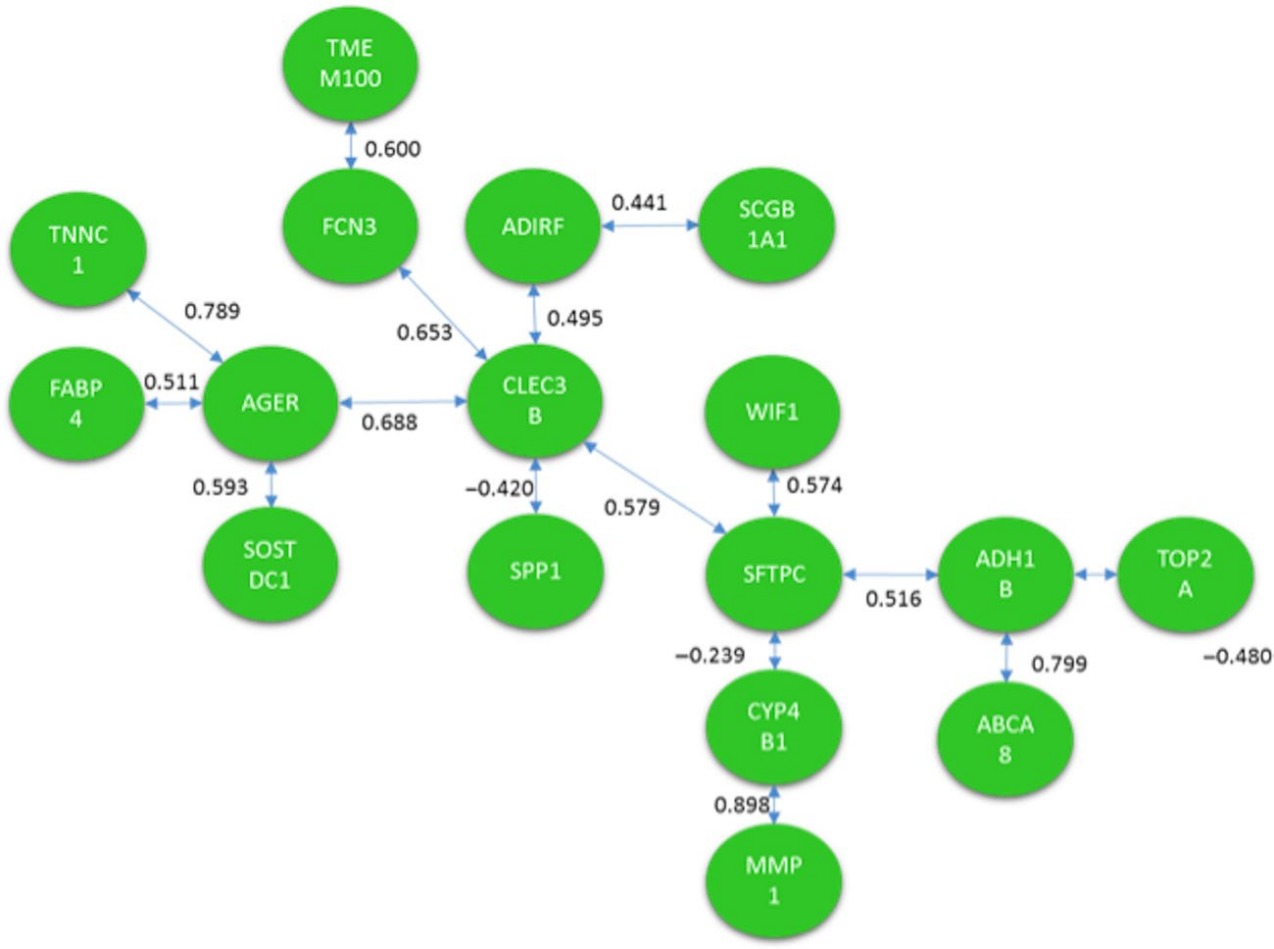
Optimal correlation tree using the minimum spanning tree formulation (healthy nonsmoker vs. cancer current smoker).

The following comparison was done between HNS versus CCS. When MCO was conducted, a total of 17 genes were identified. The TSP and MST were applied to obtain Figures 16 and 17.

**Figure 18:**
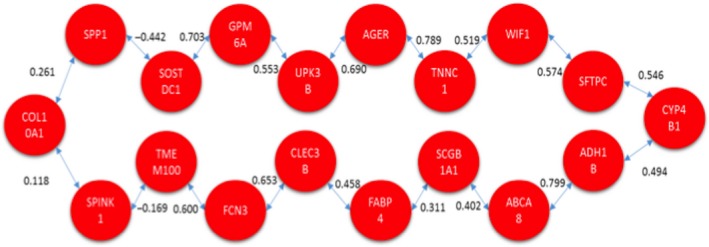
Optimal correlation optimal correlation tour using the traveling salesperson problem formulation (healthy nonsmoker vs. cancer never‐smoker).

**Figure 19:**
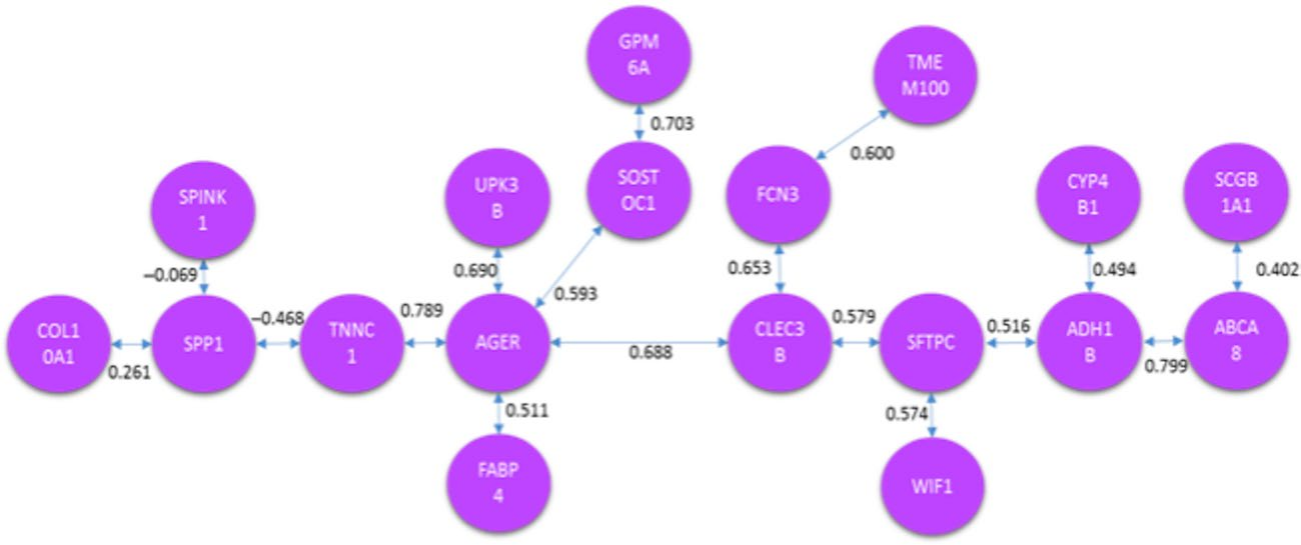
Optimal correlation tree using the minimum spanning tree formulation (healthy nonsmoker vs. cancer never‐smoker).

The HNS versus CNS comparison produced a list of 18 genes as inputs for the TSP and MST (Figs. 18 and 19)

**Figure 20:**
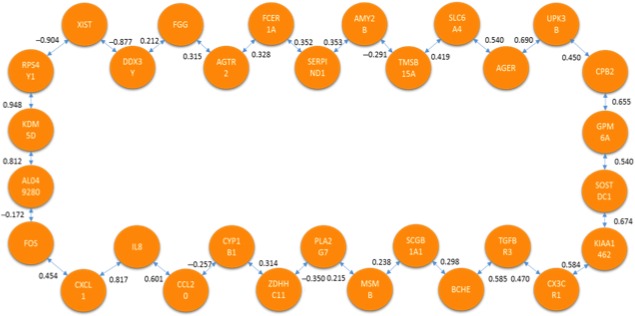
Optimal correlation optimal correlation tour using the Traveling Salesperson Problem formulation (healthy nonsmoker vs. healthy current smoker).

**Figure 21:**
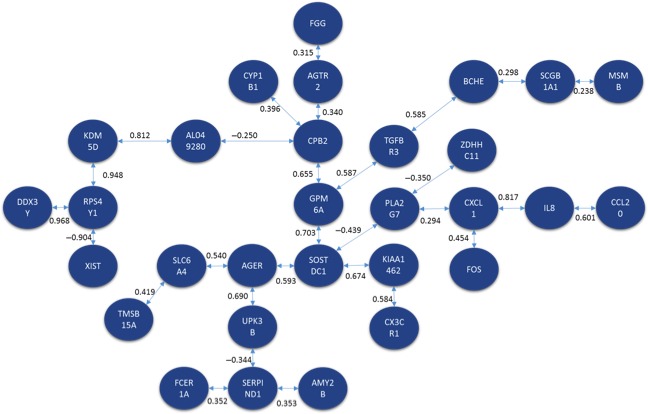
Optimal correlation tree using the minimum spanning tree formulation (healthy nonsmoker vs. healthy current smoker).

The last analysis carried out for this meta‐analysis was the comparison between HNS versus HCS. When applying the TSP and MST for this comparison, the networks were to be constructed from 30 genes of interest which heightened the number of possible solutions for both methods (Figs. 20 and 21).

The similarities that were maintained through the different analyses could prove to be biologically significant once further biological validation can be conducted. Table 4 presents relationships that were observed in several of the resulting pathways when utilizing the TSP. The relations between ABCA8‐ADH1B and TMEM100‐FCN3‐CLEC3B for example were maintained in several of the solution pathways.

**Table 4:**
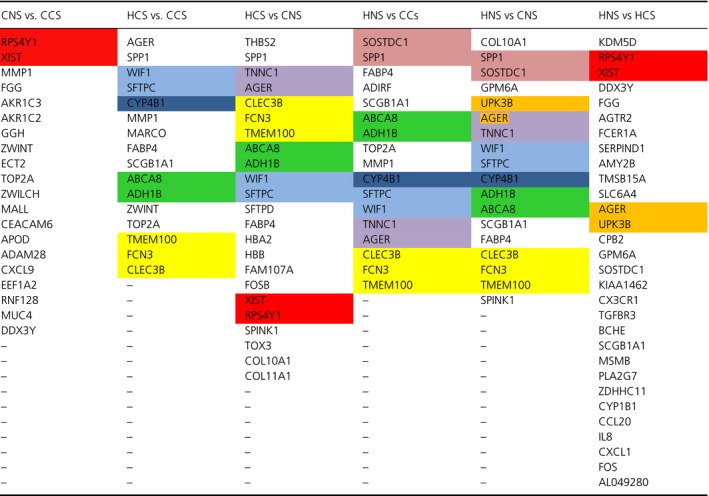
Comparison of gene relations between conditions of microarray database

Colors define blocks of correlated genes.

This comparison of signaling pathways was also done for the resulting pathways when applying the MST, Table 5 summarizes the comparison. For the MST there were also relationships maintained across different trees.

**Table 5:** Comparison of gene relations between conditions of microarray database minimum spanning tree

Comparison	Gene relationships		
	*XIST‐RPS4Y1*	*WIF1‐SFTPC‐CLEC3B‐FCN3‐TMEM100*	*AGER‐FABP4*
CNS vs. CCS	✓		
HCS vs. CCS		✓	✓
HCS vs. CNS	✓	✓	✓
HNS vs. CCs		✓	✓
HNS vs. CNS		✓	✓
HNS vs. HCS	✓		

There are some arrangements of genes common between the TSP and MST, these are included in the following Table 6.

**Table 6:** Comparison of gene relations between conditions of microarray database (TSP‐MST)

Comparison	Gene relationships (TSP‐MST)		
	XIST‐RPS4Y1	CLEC3B‐FCN3‐TMEM100	SFTPC‐WIF1
CNS vs. CCS	✓		
HCS vs. CCS		✓	✓
HCS vs. CNS	✓	✓	✓
HNS vs. CCs		✓	✓
HNS vs. CNS		✓	✓
HNS vs. HCS	✓		

TSP, traveling salesperson problem, MST, minimum spanning tree; CNS, cancer never‐smoker; CCS, cancer current smoker; HNS, healthy nonsmoker.

## Biological Evidence and Validation of Proposed Methodology

### Biological evidence for lung cancer case study

This section describes the search for existing biological evidence in literature to analyze and validate the results described in previous sections.

Based on the signaling pathways proposed by the TSP and MST formulations, public databases including KEGG and GeneCards were searched for relevant biological information. Information for the solutions for the TSP (see Fig. 4) and MST (see Fig. 5) is presented in the following Tables (Table 7 and 8). Table 7 includes information on gene relations consisting of two genes within either signaling pathway. The table indicates if any information was found for a direct relationship for the genes, or if there is an indirect relationship through one or more genes, or if no information was found for the relation. Table 8 includes the same information, but for relations including three genes.

**Table 7:** Evidence on relations consisting of two genes included in proposed signaling pathways

2 Genes						
Gene relations	Type of relationship			Traveling salesperson problem	Minimum spanning tree	Pathways
	Direct	Indirect (through various)	Not found			
*FCN3‐COL11A1*		✓		✓		
*COL11A1‐SPP1*	✓			✓		ERK signaling, phospholipase‐C pathway, PI3K‐Akt signaling pathway, degradation of the extracellular matrix, integrin pathway
*SPP1‐AGER*	✓			✓	✓	IL‐2 Pathway
*AGER‐FABP4*		✓		✓	✓	
*FABP4‐CLDN18*		✓		✓		
*CLDN18‐WIF1*		✓		✓		
*WIF1‐SFTPC*		✓		✓	✓	
*SFTPC‐CYP4B1*		✓		✓	✓	
*CYP4B1‐ADH1B*	✓			✓		Biological oxidations, metabolism, cytochrome P450 ‐ arranged by substrate
*ADH1B‐TMEM100*			✓	✓		
*TMEM100‐FCN3*			✓	✓	✓	
*FCN3‐AGER*		✓			✓	
*CLDN18‐AGER*		✓			✓	
*SFTPC‐AGER*		✓			✓	
*SFTPC‐ADH1B*			✓		✓	
*SFTPC‐COL11A1*		✓			✓	

**Table 8:** Evidence on relations consisting of three genes included in proposed signaling pathways

3 Genes						
Gene relations	Type of relationship			Traveling salesperson problem	Minimum spanning tree	Pathways
	Direct	Indirect (through various)	Not found			
AGER‐ SPP1‐COL11A1		✓		✓		IL‐2 pathway, ERK signaling, phospholipase‐C pathway, PI3K‐Akt signaling pathway, degradation of the extracellular matrix, integrin pathway
SPP1‐AGER‐SFTPC		✓			✓	
SPP1‐AGER‐FABP4		✓			✓	
SPP1‐AGER‐FCN3		✓			✓	
SPP1‐AGER‐CLDN1		✓			✓	IL‐2 pathway, integrin pathway

Several gene relations were identified to have either a direct or indirect connection. SPP1 and AGER (also known as RAGE) is one example of a direct relation that has been previously identified and forms part of the IL‐2 pathway. The COL11A1 and SPP1 relation is also documented within several known pathways as described in Table 7. Various other gene relations had evidence of being related to one another through other genes. In addition, the relation including AGER, SPP1, and COL11A1 is documented within various known pathways (see Table 8).

The gene relations included in our results can be categorized into two classifications, the first being gene relationships connected to lung cancer already reported, serving to validate our methodology. The second classification includes gene relationships involved in other types of cancer, which offer the greatest possibility for discovery. In the first type of classification, genes COL11A1, SPP1, AGER, WIF1, SFTPC, FABP4, and CLDN18 are included. Existing literature has previously established the change in expression in lung cancer for several of these genes, as well as being part of the correlated pathways between known driver genes (K‐Ras and EML4‐ALK) and tumor suppressor genes (RAR‐beta, FHIT, RASSF1, INK4a/ARFand p53). The correlated pathways between driver genes, tumor suppressor genes, and the genes of the first classification are as follows: COL11A1‐SPP1 through ECM receptor/PI3K‐Akt pathway, AGER through IL‐2 and HGMB1 pathway, WIF1‐SFTPC through WNT and WNT mediated beta catenin signaling pathway, CLDM18 through its function in tight junction on pathways.

The second classification of genes includes FCN3, ADH1B, TMEM100, and CYPB1. The expression changes of these genes have been reported for different cancer pathways. These include complement pathway, chemical carcinogenesis, tumor suppression, and cytochrome P450 inflammatory responses respectively. Furthermore, all the genes have been correlated with inflammatory responses. These commonalities infer a possible relation to lung cancer pathways.

### Biological evidence for meta‐analysis results

As described in Section “Meta‐analysis”, diverse gene relations could be observed throughout several of the comparisons between the different groups (see Fig. 9). Similarly with the previous section, a search for existing biological evidence was conducted based on the gene relations that were maintained in the TSP (see Table 7).

Sun et al. 40 analyzed microarray data containing information on Parkinson's disease. They identified a total of 10 distinctly differentially expressed genes, including RPS4Y1 and XIST, genes that were connected in our proposed TSP signaling pathway, presenting the possibility that the expression of these genes is coordinated (for men). According to their analysis, they concluded that RPS4Y1 and XIST, along with PRKAG2 and DLG1, can be regarded as gene biomarkers for Parkinson's disease 40.

A relation between the expressions of ABCA8 and ADH1B, was reported by Liu et al. 41, in their study of ovarian cancer progression, where it was found that ABCA8 and ADH1B were part of six genes that were downregulated in the condition. Through their analysis they concluded that high expression of ABCA8, along with ALDH1A2, might predict poor outcome in terms of survival. In addition, the report mentions that ADH1B and ALDH1A2 might be associated with drug resistance 41. Based on their conclusions, both ABCA8 and ADH1B have a role in ovarian cancer, which could possibly be similar in lung cancer according to our results.

**Figure 22:**
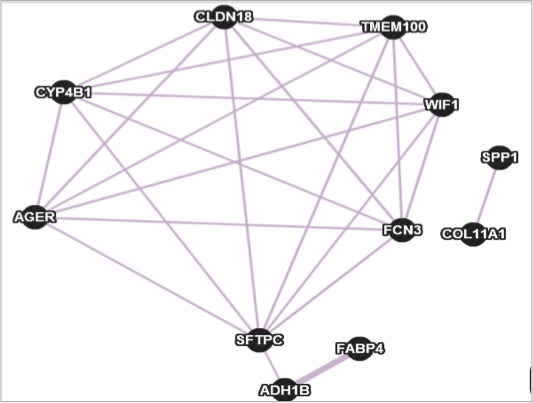
Coexpression network constructed by geneMANIA.

The results for the PANTHER 42 Molecular Function Ontology tool (Fig. 22) provide information about common molecular functions that may be affected by the expression change in the genes selected in the different comparisons. Molecular functions possibly affected in the seven comparisons (including the global comparison) are: binding, structural molecule activity, catalytic activity, and transporter activity. When comparing the control and cancer groups for nonsmokers and current smokers, the common molecular functions are: translation regulator activity, binding, structural molecule activity, catalytic activity, and transporter activity. The common molecular functions between control and cancer for current smokers, and control and cancer for nonsmokers are: binding, receptor activity, structural molecule activity, catalytic activity, and transporter activity. Even as some of the genes change from list to list, there are common molecular functions that are maintained across the different comparisons, including the comparison between nonsmokers and current smoker controls.

### Comparison to existing software

There are several methods or tools for identifying and constructing signaling pathways. GeneMANIA 43 was the tool selected for comparison with the methodology described in this work. It is a tool that has a web interface for generating hypotheses about gene function, analyzing gene lists, and prioritizing for functional analysis 44.

GeneMania uses publicly available databases 45. This includes coexpression data that is collected from Gene Expression Omnibus, physical and genetic interaction data from BioGRID, and predicted protein interaction data from the orthology Interologous Interaction Database (I2D). Pathway and molecular interaction data from Pathway Commons are also included, which contains data from BioGRID, Memorial Sloan‐Kettering Cancer Center, Human Protein Reference Database, and HumanCyc, among others 45.

In GeneMANIA, a researcher must provide a predetermined list of genes of interest. The program then extends the list with genes that are functionally similar or have shared properties with the initial query genes. The output is in the form of a display for the interactive association network, which illustrates the relations among the genes and data sets 45.

GeneMANIA is based on a heuristic method that builds a composite functional association network by integrating multiple functional association networks and predicts gene function 46. The constructed composite network is a weighted sum of individual data sources. Each edge in the composite network is weighted by the corresponding individual data source. Given the composite network, GeneMANIA uses label propagation to score all genes not in the query gene list. The scores are used to rank the genes. The score assigned to each gene reflects how often paths that start at a given gene node end up in one of the query gene nodes and how long and heavily weighted those paths are 45.

The list of 11 genes in Table 3 from our lung cancer case study was used to compare the TSP and MST methodologies and GeneMANIA.

Figure 22 is the resulting overlap of three networks that GeneMANIA constructed to relate or connect one gene to another from our original query list. Additionally GeneMANIA included 20 additional genes to the query list in order to construct the networks. The maximum total of additional genes that the tool can include is determined by the user from a range of 0 to 100, with a default value of 20.

The network that generated the largest number of relationships and the largest network connecting all of the original eleven genes was that of the coexpression factor. In total there are 24 connections that form part of the network that GeneMANIA constructed. It must be noted that GeneMANIA was unsuccessful in including all genes in the list. SPP1 and COL11A1 were not connected to the rest of the genes.

**Figure 23:**
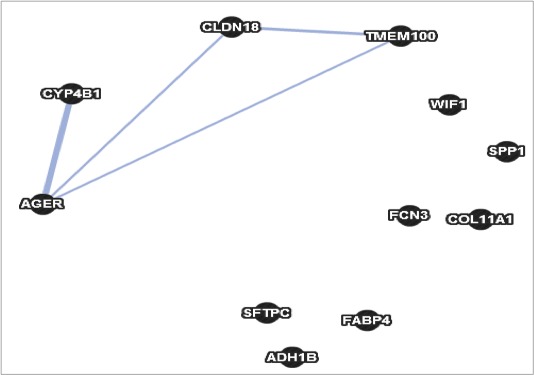
Colocalization network constructed by geneMANIA.

The second network constructed by GeneMANIA containing some genes of our query list is the category of colocalization. As can be seen in Figure 23, GeneMANIA did not include five of the eleven genes as part of the resulting network. In this category GeneMANIA only connected four genes from the query list with a total of four connections. It was not successful in directly relating all the genes to each other (Fig. 23).

**Figure 24:**
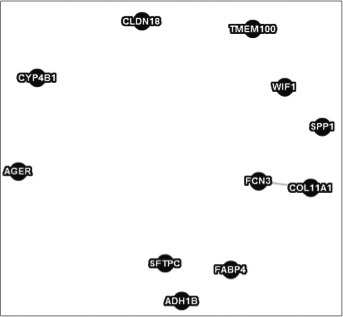
Shared protein domains network constructed by geneMANIA.

The third and last network generated by GeneMANIA can be seen in Figure 24. This network is categorized as genes that share protein domains. This network only links two of the eleven genes. As in the other categories it was unsuccessful in linking all of our original query list of genes.

In our case study for lung cancer, GeneMANIA generated three networks based on three separate categories. Only in the first category of coexpression, GeneMANIA was successful in establishing a network linking all eleven of the genes of interest. None of these three networks generated by GeneMANIA was in the category of pathways.

GeneMANIA relies on published analysis to generate networks; our methodology does not have this dependence for generating possible signaling pathways. Therefore our method possesses an interesting potential for determining previously undiscovered pathways or gene relations important in a disease, in this case lung cancer. Also, due to its deterministic nature, our methodology is able to obtain a global optimal solution with either the TSP or MST. In addition, both methods were capable of obtaining solutions that include all genes from a list.

## Conclusions

Identifying cancer biomarkers is an important step in the diagnosis, prognosis, and prevention of this disease; and determining how these biomarkers are related or interact is just as important. A signaling pathway among these biomarkers is, then, a worthy aim. Uncovering signaling pathways for potential biomarkers could further the understanding of the origins and the evolution of cancer.

As a first approach to structure a network of potential biomarkers, the linear correlations that exist among these genes are used. Once the network of a preselected list of potential biomarkers is constructed, the TSP can be applied to obtain an optimal sequence that maximizes the linear correlations. This sequence represents the potential signaling pathway. In Section “Case study: lung cancer” biological information was gathered supporting the potential of our methodology and of the important gene relations in our proposed signaling pathway.

In addition, the MST formulation is used here as an alternative method to the TSP to discover a signaling pathway from the same list of preselected potential biomarkers. The MST provides an optimal tree, a representation that must be contrasted to that of TSP's tour.

In Section “Traveling Sales Problem” of this article includes the exploration for biological evidence to assess the resulting signaling pathways constructed when comparing the different conditions of the microarray database described in Section “Case study: lung cancer”. Several groupings of genes were common throughout the different comparisons, suggesting a possible biological significance worthy of experimental biological validation. Future work, will investigate consistency on gene selection and network structuring using different databases.

Three important aspects are novel in this work: (1) the joint analyses of different groups of lung cancer states reveal new correlations, biologically evidenced, and previously undocumented; (2) computation of the correlation coefficients from expression differences leads to an effective use of network optimization methods; and (3) the methods yield mathematically optimal correlation structures: no other configuration is better correlated with the available information.

## Conflict of Interest

None declared.
